# Fuel Effects on Aviation Engine Emissions: A Chemical Reactor Network Modeling Study

**DOI:** 10.1021/acs.energyfuels.5c05727

**Published:** 2026-02-20

**Authors:** Dario Lopez-Pintor, James MacDonald, Elkin Ramirez-Correa, Jose Maria Garcia-Oliver, Raul Payri, Pedro Marti Gomez-Aldaravi, Pénélope Leyland

**Affiliations:** 1 1105 Sandia National Laboratories, Livermore, California 94550, United States; 2 CMT - Clean Mobility & Thermofluids, Valencia, Valencia 46022, Spain; 3 Advanced Engineering Design Solutions (AEDS), 1921 Martigny-Croix Switzerland, and Tribology and Interfacial Chemistry Group, IMX, EPFL, Lausanne 1015, Switzerland

## Abstract

The present study investigates the formulation of surrogates for Jet-A and sustainable aviation fuel (SAF) by means of a chemical reactor network (CRN) to predict emissions in gas-turbine combustors. The modeling framework is used to analyze the effects of fuel composition, specifically the replacement of Jet-A with SAF as well as the substitution of aromatics in Jet-A with cycloalkanes. This approach enables the examination of the effects of the fuel class and molecular structure independently of global exhaust emission trends. A comprehensive chemical kinetic mechanism incorporating 8478 species and 33,318 reactions was used to model jet fuel surrogates. This mechanism, validated against ignition delay times, laminar flame speeds, and extinction strain rates, accurately predicted combustion characteristics for iso-cetane and iso-dodecane, key components of SAF. Surrogate fuels for Jet-A and alternative fuels were formulated targeting critical properties such as density and cetane number. Surrogate simulation results show strong alignment with experimental data on ignition delay and flame speed, further confirming the reliability of the surrogates. The CRN model was developed using the CFM56 engine data and validated against the International Civil Aviation Organization emission benchmarks. After that, two different fuel replacement scenarios, involving Jet-A, are investigated. In the first one, Jet-A is compared to 100% SAF, which helps assess expected differences in combustion performance if a fully renewable fueling supply is followed. Results show that NO_*x*_ emissions are unaffected by SAF, aligning with previous experimental studies. Polycyclic aromatic hydrocarbons (PAH) decrease by 93% for SAF compared to Jet-A, while Jet-A produces more CO due to its aromatic content. Substituting aromatics with cycloalkanes in Jet-A reduces PAH emissions by up to 96 or 92%, depending on whether all aromatics are replaced or only diaromatics. In a second scenario, aromatics are removed from conventional Jet-A and replaced with cycloalkane species, which can still hold the swelling properties needed by the fueling system. In terms of combustion, cycloalkane substitution leads to slightly increased CO emissions and reduced flame temperatures. These results demonstrate the potential of SAFs and cycloalkanes in reducing soot precursors while maintaining a highly similar performance in the combustion process. Overall, the proposed CRN framework provides a good example of how a predictive and computationally efficient tool can help in the early evaluation of alternative aviation fuels under realistic gas-turbine combustor conditions.

## Graphical abstract

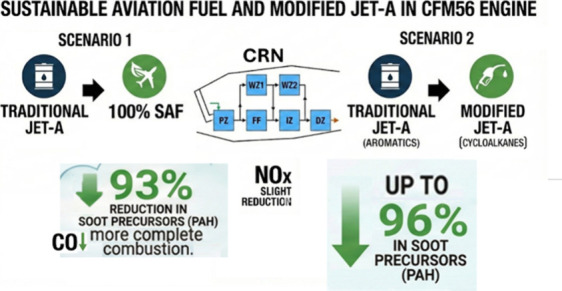

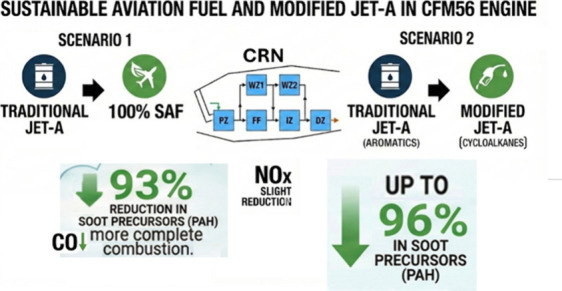

## 1 Introduction

In 2021, the transportation sector reached a critical milestone, as the airline industry, guided by the International Air Transport Association (IATA), committed to achieving climate neutrality by 2050. This sector accounts for a quarter of the world’s greenhouse gas emissions, with aviation alone contributing 14% to the total transportation sector.^1–4^ Sustainable aviation fuels (SAFs) have emerged as a critical solution. These renewable liquid fuels, with very low levels of aromatic content and no sulfur in their compounds, mimic traditional aviation fuel and are compatible with existing aircraft technology and infrastructure.^5,6^ The complete transition to SAFs could significantly reduce the annual mean net radiative forcing of contrails by up to 44%, cut the nonvolatile particulate matter (PM) emissions index by up to 70%, and provide at least 80% less CO_2_ emissions compared to traditional aviation fuel.^7–9^ This move toward alternative fuels marks a significant step in reducing aviation’s environmental footprint and aligning with international climate goals. Despite their higher production costs, the push for their adoption is supported by emerging policies and voluntary industry purchases.^10–12^ The escalating environmental impact of aviation emissions, coupled with the projected aviation activity by 2050,^13^ highlights the urgent need for comprehensive methodologies in SAF surrogate formulation, validation, and advanced predictive modeling to estimate particulate matter and gaseous emissions from these fuels. The National Jet Fuels Combustion Program (NJFCP) has significantly contributed to SAF advancements by establishing a robust framework for evaluating new fuel formulations that replicate critical properties.^14^ This framework facilitates the assessment of similarities to conventional fuels in combustion and operability tests, providing a reliable foundation for SAF evaluation.^15^

The chemical composition of the fuel influences nonvolatile PM emissions, such as soot,^16,17^ with experiments by Richter et al. showing that fuels containing more aromatics or highly branched alkanes have a higher propensity to form PM. Polycyclic aromatic hydrocarbons (PAHs) are precursor particles to soot both experimentally and for modeling purposes.^18–20^ Most research in this area is experimental, focusing on the blending of conventional aircraft fuel with SAFs. At the engine level, Bulzan et al.^21^ measured emissions from an auxiliary power unit (APU) using a Fischer–Tropsch (FT) fuel made from coal and found a considerable reduction in particulate emissions primarily due to the almost complete absence of aromatics. Cain et al. also presented the dependence of aromatic formation on fuel composition, testing conventional aviation fuel and iso-paraffinic kerosene. The authors also demonstrated that a low aromatic content in the fuel reduces the formation of benzene and toluene, which is important since these species are known precursors of PAHs and subsequent soot.^22^ Schripp et al. evaluated the impact of alternative jet fuels on engine exhaust composition and reported that the measured particle emission indices showed a reduction of up to 50% (number) and 70% (mass) for two alternative jet fuels at low power settings in comparison to the reference fuels.^23^ These results are consistent with those of Corporan et al., who demonstrated how lower aromatic content in alternative jet fuels, such as those derived from Fischer–Tropsch processes, leads to significantly reduced particulate matter emissions compared to conventional jet fuels like JP-8.^24^

Overall, these findings suggest a robust and consistent consensus across engine-scale experiments, indicating that SAF deployment results in a substantial reduction in soot-related emissions. This behavior has been observed not only in ground-based engine and APU tests but also in transient operating conditions and during dedicated in-flight measurement campaigns, covering a wide range of engine power settings and operating regimens.^22,25^

In these studies, the reduction in particulate emissions is primarily attributed to the lower aromatic content and higher hydrogen-to-carbon (H/C) ratio of SAFs.^23^ Gaseous emissions, such as NO*_x_*, are generally reported to remain largely unchanged, and CO variations are minor and strongly dependent on operating conditions.^26^

According to ASTM D7566, jet fuel requires a minimum content of aromatic compounds to ensure sufficient seal swelling in aircraft and fuel circulation systems.^24,27^ Nevertheless, evidence supports that species such as cycloalkanes can replace aromatics in the fuels to perform the important seal-swelling function, thereby reducing the effect of the aromatics in producing PAH.^28^ Kinetic studies^29^ have shown that substituting aromatics with cycloalkanes can influence fuel reactivity, ignition delay, and NTC behavior in HEFA-based SAFs, but the implications of such substitutions on combustor-scale combustion performance and pollutant formation, remain largely unexplored.

Within the previous context, the development of 0D/1D models incorporating detailed jet fuel chemistry to predict emissions has become a valuable tool for designing emission reduction strategies.^26^ Approaches such as chemical reactor networks (CRN) balance computational efficiency and accuracy while still delivering relevant information to understand exhaust emissions depending on fuel composition. Recent studies, such as those by Villete et al. and Khodayari et al., have highlighted the versatility of CRN in predicting the emission of minor species concentrations and its ability to model fuel-specific kinetics and transport phenomena.^30,31^ Additionally, Yi et al. demonstrated the use of 0D simulations with advanced soot models to analyze how fuel chemical structures influence soot formation, a critical aspect of emissions that varies significantly between SAF and Jet-A.^32^ These insights are crucial for validating CRN models against real-world data, particularly when assessing new fuel compositions versus conventional ones.

Table 1 summarizes literature studies dealing with CRN applications to aeroengine exhaust pollutant analysis. This modeling approach has proven instrumental in the early stages of aircraft design. For instance, Saboohi and Ommi addressed emission prediction in the conceptual design phase of aircraft engines using an augmented CRN. In their work, the authors emphasized the need for comprehensive models that can quickly provide data during the early design phases, which is crucial for informed decision-making in the development of cleaner and more efficient engines.^35^ In this context, CRN is defined as a tool that not only models combustion but also integrates detailed chemistry, reinforcing its relevance in emissions research. Similarly, Bisson et al. and Starik et al. have utilized CRNs to model not only gaseous emissions but also aerosol precursors and particle formation, providing a comprehensive understanding of aviation engine emissions under various operating conditions.^34,33^ Given the high expenses associated with developing new SAF formulations, CRN modeling serves as an invaluable tool, enabling the assessment of fuel viability in an early stage, cost-effective manner both economically and computationally.

**Table 1:** Summary of Previous CRN-Based Aviation Emission Studies

reference/year	study focus/contribution	emissions studied/fuel type	aromatics—cycloalkane substitution	chemical mech
**Moniruzzaman** **and Yu** ^26^	aircraft engine emission model based on a parcel-based CRN with mixed and unmixed zones, coupling detailed chemistry and soot microphysics; validated across ICAO thrust settings, limitations in simultaneously predicting CO and NO*_x_*	NO_*x*_, CO, soot precursors/conventional Jet-A	no	359 chemical species and 2657 reversible reactions
**Starik et al.** ^33^	engine-scale emission modeling assessing emission sensitivity to operating regimes, capturing regime-to-regime variability across different engine conditions	NO_*x*_, CO/conventional Jet-A	no	no data
**Bisson et al.** ^34^	prediction of gaseous aerosol precursors and particles using a CRN coupled with soot/aerosol models and CFD-informed residence times; applied across ICAO thrust settings, with limitations in simultaneously predicting CO and NO*_x_*	NO_*x*_, CO, PM precursors/conventional Jet-A	no	no data
**Saboohi** **and Ommi** ^35^	emission prediction for conceptual aircraft engine design using an augmented CRN with simplified chemistry, suitable for early stage design and focused on design-oriented emission trends, with limited detailed validation	NO_*x*_, CO/conventional Jet-A	no	Ranzi: 484 species and 19,341 reactions
				GRI 3.0:53 species and 325 reactions
				Kollrack: 21 species and 30 reactions
**Zhang et al.** ^36^	prediction of NO*_x_* and CO in low-emission concentric-staged combustors using simple and detailed CRN models coupled with RANS CFD; good agreement with empirical correlations, identifying pilot and recirculation zones as main NO*_x_* sources	NO_*x*_, CO/conventional aviation fuel	no	374 step elementary reactions and 82 species
**Khodayari** **et al.** ^31^	review of CRN-based pollutant emission prediction, summarizing studies using flow-field pattern-based CRN construction; while predictions are generally satisfactory, approach is highly case-dependent and requires adaptation to different combustor geometries and flow regimes	pollutant emissions (general)/various aviation fuels	no	2-to-8 reactions and 5-to-8 species, depending on fuel type and required level of detail
**Villette et al.** ^30^	simplified CRN for aeroengine combustor design, using geometry and equivalence ratios, efficiently capturing ICAO trends with good NO*_x_* accuracy, CO/UHC accuracy varies	NO_*x*_, CO, UHC/conventional Jet-A	no	991 step elementary reactions and 91 species
**Garcia-Oliver et al.** ^37^	CRN model for aviation gas turbines, topology-informed and tailored to combustor configuration, demonstrating applicability to modern combustors	NO_*x*_, CO/conventional Jet-A	no	991 step elementary reactions and 91 species

While CRNs have been extensively used in the past to study aerocombustor emissions, Table 1 also demonstrates that most of the previous studies focused on traditional aviation fuel, while the fuel effects of SAFs have not been investigated in detail, particularly from a combustor-scale and chemistry-resolved perspective. Similarly, the analysis of the effects of substituting aromatics with cycloalkanes has not been dealt with from a perspective such as the one presented here. Moreover, most of the previous works utilized relatively simple chemical mechanisms and surrogates with a less comprehensive description of fuel chemistry, limiting their ability to capture fuel-specific soot precursor formation pathways. The present paper proposes a model that employs a chemical reactor network approach, coupled with a detailed chemical kinetic mechanism and surrogate formulations for various aviation fuels, to predict gaseous pollutant formation in the combustor of a CFM56 jet engine. Soot production in the engine is analyzed based upon PAH predictions from the detailed model. The present approach moves beyond exhaust-based trend analysis toward a mechanistic interpretation of fuel effects within the combustor, which explicitly isolates the role of the fuel class and molecular structure.

The proposed modeling workflow has been used to explore two scenarios of interest within the current interest of the aeroengine industry: The first scenario compares traditional Jet-A and three different SAF surrogates to quantitatively evaluate the potential changes in pollutant prediction induced by the 100% SAF strategy compared to conventional Jet-A fuels, which cannot be within certification limits nowadays but it might be in the future. The second scenario evaluates the changes in emission from a Jet-A fuel when aromatics are replaced by three types of cycloalkanes, allowing a systematic decoupling of the aromatic content from overall fuel composition effects. Overall, this research presents a modeling approach to evaluate different fuel formulations at an early design stage, which could potentially contribute to reducing the aviation sector’s impact on air quality and global greenhouse gas emissions.

## 2 Methodology and Models

This section presents the methodology used to model jet fuel combustion, including the CRN model description, the selection of the detailed chemical mechanism, and the formulation of the surrogate fuels. All calculations needed were performed with an ANSYS CHEMKIN-PRO.

### 2.1 Chemical Reactor Network Formulation

Figure 1 illustrates the configuration of the CRN selected for modeling the gas-turbine combustor, which is derived from a previous study by Garcia-Oliver et al.^37^ This configuration, consisting of a set of core zones surrounded by wall zones and with air flows flowing from the boundaries of the combustor to the center, has been widely implemented by many authors.^30,38–40,37^ The CRN configuration is composed of a set of perfectly stirred reactors (PSRs) that represent different zones in the combustion chamber:

- A primary zone (PZ) with a known rich equivalence ratio obtained from computational fluid dynamic (CFD) simulations from the literature.^36^
- A flame front (FF) zone, where the main reaction is assumed to occur at stoichiometric conditions.
- An intermediate zone (IZ), where combustion reaction is completed, and a dilution zone (DZ), where the final amount of air is introduced to control outlet temperature before the gas turbine.
- Two reactors (WZ1 and WZ2) defined as wall zones, which receive the air mass flow coming through the wall holes of the combustor chamber.

**Figure 1:**
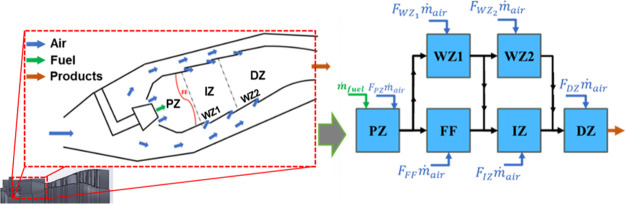
CRN configuration.

Following the original approach,^37^ the flow distribution parameters within the network together with the length of the flame zone FZ have been calibrated for the standard operating conditions (takeoff, climb, approach, and idle) in an iterative process to achieve the best alignment with the temperature distribution obtained in CFD by ref 36. Table 2 presents the operational conditions at the combustor inlet, which include the mass flow of air *ṁ*_air_, fuel flow rate *ṁ*_fuel_, and the air inlet pressure *P*_in_ as well as temperature *T*_in_.^41^ The chemical mechanism used for the study is presented in Section 2.2, and the fuel surrogate used for Jet-A is described in Section 3.1. The calibration parameters resulting for this process are included in the Supporting Information, Table S1, together with the geometrical parameters, Table S2, which are determined based on the findings presented by Saboohi et al.^41^

**Table 2:** Operating Conditions at the Combustor Inlet^41^

operating conditions for CFM56 7B27/B1F				
parameter	takeoff	climb	approach	idle
*ṁ*_air_[kg/s]	44.52	39.1	20.87	12.12
*ṁ*_fuel_ [kg/s]	1.28	1.04	0.349	0.119
φ [-]	0.42	0.39	0.25	0.14
*P*_in_ [MPa]	2.9	2.48	1.13	0.559
*T*_a,in_ [K]	800	764	613	505

### 2.2 Selected Chemical Kinetic Mechanism

A comprehensive chemical kinetic mechanism for jet fuel and diesel fuel surrogates developed by Lawrence Livermore National Laboratory (LLNL) was used in this paper.^42,43^ The mechanism consists of 8478 species and 33,318 reactions and is a combination of multiple submechanisms for *n*-alkanes, iso-alkanes, cycloalkanes, aromatics, and olefins. The mechanism for diesel surrogates proposed by Wang et al.^42^ was used as core chemistry, with 2-methyl alkane chemistry from Sarathy et al.^43^ PAHs were modeled up to cyclo-penta pyrene (C18H10) using the mechanism developed by Kukkadapu et al.,^44^ and detailed nitrogen oxide (NO_*x*_) chemistry was modeled with the mechanism of Glarborg et al.^45^ The mechanism has been validated against ignition delay time, laminar flame speed, and reactor speciation measurements elsewhere,^42,44,46–48^ and an additional validation for selected species of interest for jet fuel is included here.

The performance of the mechanism in modeling the combustion of iso-cetane (2,2,4,4,6,8,8-heptamethyl nonane) and iso-dodecane (2,2,4,6,6-pentamethyl heptane), two highly branched iso-paraffins representative of those typically found in SAF, was assessed by simulating ignition delay times, counter-flow extinction strain rates, and laminar flame speeds. These species were selected for an additional validation of the mechanisms because the alcohol-to-jet fuel produced by Gevo for the NJFCP (C1) and tested in this work is composed almost exclusively of highly branched C12 and C16 iso-paraffins.^49^ On the other hand, the Fischer–Tropsch iso-paraffinic kerosene tested in this study is mainly composed of iso-paraffins with one-to-four branches, and the chemical mechanism has been extensively validated already for 2-methyl alkanes and other lightly branched iso-paraffins.^42^

Shock tube experiments from the literature were simulated using a closed, adiabatic, constant-volume, and homogeneous reactor in which the conditions behind the reflected shock wave wereimposed as initial conditions of the simulations, and ignition delay was modeled as the time of themaximum temperature rise rate (d*T*/d*t*). Rapid compression machine experiments from the literature were simulated by imposing the conditions at the end of compression as the initial conditions of the simulation. For those cases where a quantification of heat transfer losses during the ignition delay time was available, they were incorporated in the simulations by imposing an effective volume profile derived from the experiments. For cases in which heat transfer information was unavailable, simulations were considered adiabatic. For this reason, comparisons of rapid compression machine experiments and simulations presented in this article should be analyzed with caution. Figure 2 shows the ignition delay of iso-cetane from the experiments of Won et al.^49^ and Oehlschlaeger et al.^50^ and the corresponding simulations using the mechanism described above. More specifically, Figure 2a shows the ignition delay of stoichiometric iso-cetane/air mixtures at 40 bar and Figure 2b shows the ignition delay of iso-cetane/air mixtures at 10 bar and for three different equivalence ratios (φ = 0.5, 1.0, and 1.5). The mechanism reproduced the experiments with high accuracy, including the negative temperature coefficient (NTC) zone of the fuel (Figure 2a). Figure 3 shows the ignition delay of iso-dodecane from the experiments of Won et al.,^49^ Ruozhou et al.,^46^ and Mao et al.,^51^ and the corresponding simulations from this work. Results at stoichiometric conditions and different pressures are shown in Figure 3a, and results at 20 bar and different equivalence ratios are shown in Figure 3b. In general, the mechanism shows very good agreement with the experiments with larger deviations at lean conditions. The level of accuracy shown during the NTC regime at lean conditions is similar to that reported by other authors,^51^ suggesting that further refinement of the chemistry is required (which is not within the scope of the present study). Nevertheless, the simulations performed in this study comprise continuous combustion under steady-state conditions in a rich-burn combustor, where autoignition chemistry during the NTC regime under lean conditions plays a secondary role. Considering the good agreement between simulations and experiments at φ = 1, 1.5, and 2, the model is considered to be valid for the present study.

**Figure 2:**
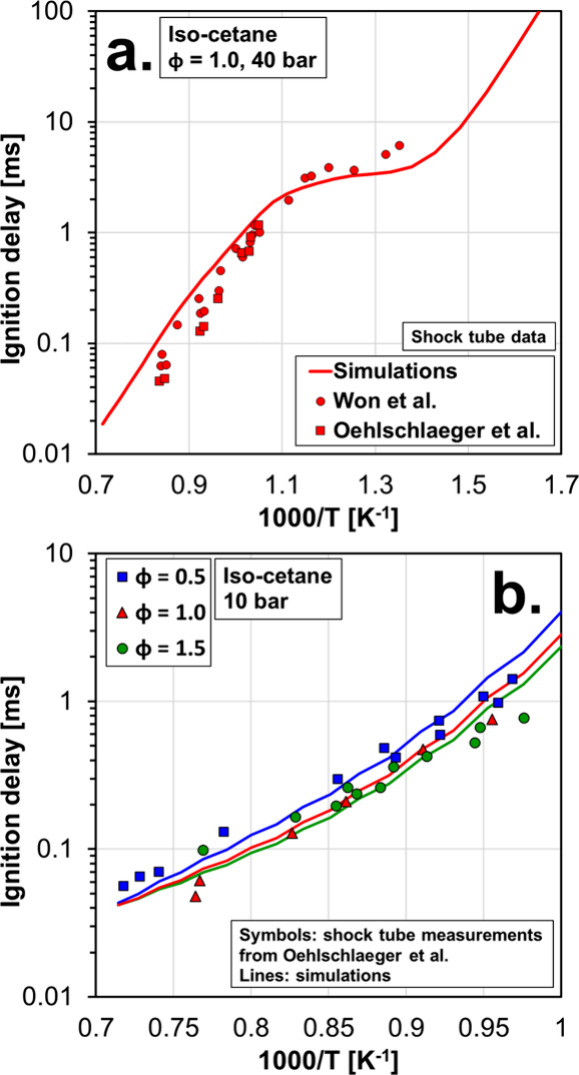
Ignition delay times of iso-cetane measured in shock tube facilities from Won et al.^49^ and Oehlschlaeger et al.^50^ and corresponding simulations with the detailed mechanism used in this work. Results are grouped by the operating pressure, with corresponding to (a) 40 bar and (b) 10 bar cases.

**Figure 3:**
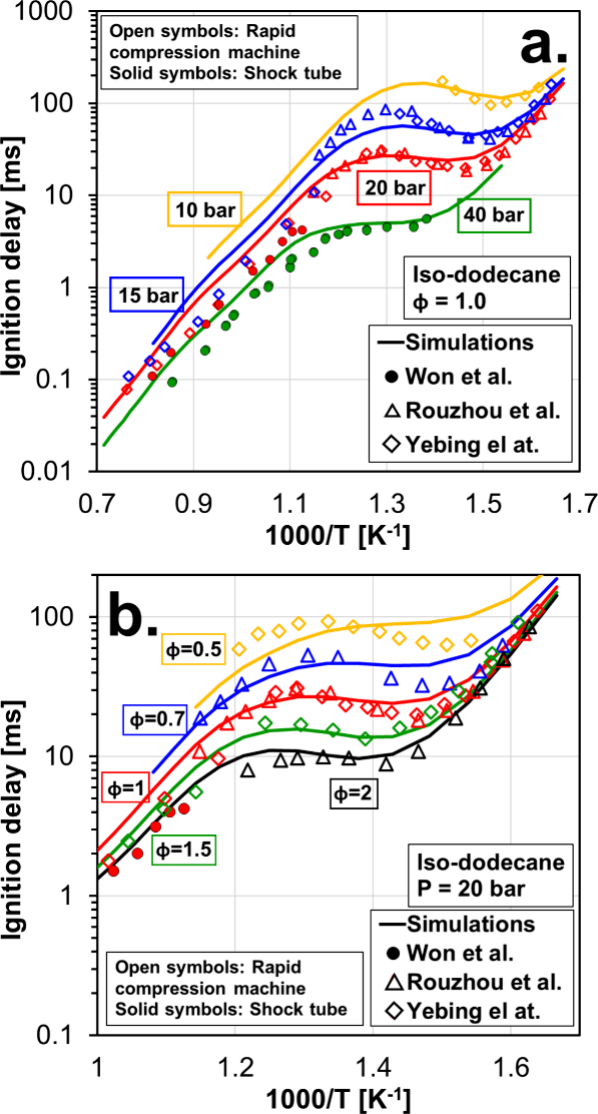
Ignition delay times of iso-dodecane from Won et al.,^49^ Fang et al.,^46^ and Mao et al.^51^ and corresponding simulations with the detailed mechanism used in this work. Trends with temperature are shown in panel (a) for a parametric variation of the pressure and in panel (b) for a parametric variation of the equivalence ratio.

Extinction strain rate measurements in counter-flow configuration were simulated using an opposed-flow flame 1D reactor in which the boundary conditions of the experiments of Won et al.^49^ were imposed, and results are shown in Figure 4 for iso-cetane (panel (a)) and iso-dodecane (panel (b)). Mixture-averaged transport properties were used to compute the diffusion coefficients and fluxes, and thermophoresis was included in the calculations. The fuel and oxidizer velocities were adjusted in the simulations until flame extinction was achieved, from which the corresponding extinction rate (*a*_E_) was calculated consistently with the approach of Won et al. as follows:

$$a_{E}=\frac{2U_{\mathrm{oxidizer}}}{L}\left(1+\frac{U_{\mathrm{fuel}}}{U_{\mathrm{oxidizer}}}\sqrt{\frac{\rho_{\mathrm{fuel}}}{\rho_{\mathrm{oxidizer}}}}\right)$$

where *U* represents flow velocity at the nozzle exit, *L* is the distance between fuel and oxidizer nozzles, and ρ represents density. Experiments and simulations were performed at various fuel mass fractions, adjusted by adding nitrogen to the fuel stream (see ref 49 for details on the experimental conditions). The mechanism reproduces the experimental trends with a high accuracy. Deviations between experiments and simulations are generally within the uncertainty of the experiments for iso-dodecane, but simulations overpredict the extinction strain rate of iso-cetane. Therefore, extinction simulations of fuels with high iso-cetane content should be taken with caution.

**Figure 4:**
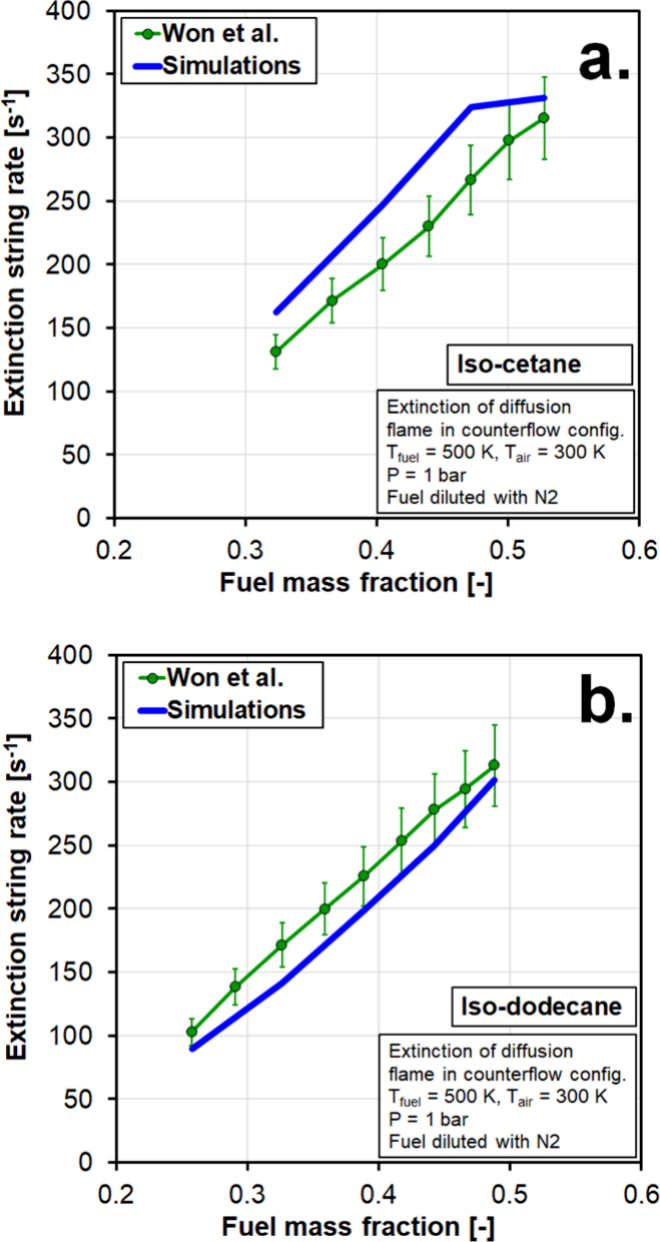
Extinction strain rate for iso-cetane (a) and iso-dodecane (b) in a counter-flow reactor configuration. Experiments of Won et al.^49^ are compared against 1D simulations.

Laminar flame speed measurements of iso-cetane from Li et al.^52^ were simulated using a 1D flame tube reactor, where the boundary conditions from the experiments were imposed and the flame speed was defined as the inlet velocity that allows the flame to stay in a fixed location. Results are shown in Figure 5. Diffusive effects were calculated by using the mixture-averaged transport properties, and thermophoresis effects were included in the calculations. The results obtained with the model match those of the experiments with deviations within the experimental uncertainty reported by Li et al. Unfortunately, the authors could not find laminar flame speed measurements of iso-dodecane in the literature.

**Figure 5:**
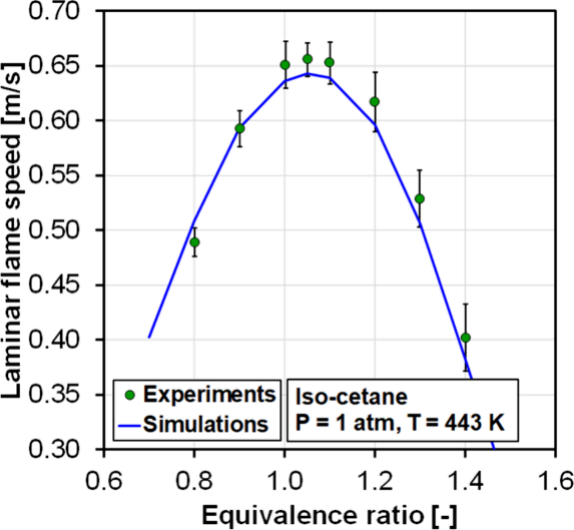
Laminar flame speed of iso-cetane for an equivalence ratio sweep at 1 atm and 443 K. Experiments from Li et al.^52^ are compared against simulations.

### 2.3 Formulation of Surrogate Fuels

The methodology for surrogate formulation was based on previous work.^53,54^

- An initial surrogate definition was chosen based on the composition of the target fuel (from comprehensive two-dimensional gas chromatography, GCxGC, measurements), considering the distribution of hydrocarbon classes and the carbon number distribution within each hydrocarbon class. The species used for the formulation of the surrogate were selected based on chemical kinetic mechanism availability and abundance in the real fuel (selected species are representative of each hydrocarbon class present in the target fuel considering not only the carbon number but also the molecular structure). It should be noted that fuel cost or blending feasibility in a laboratory environment was not considered in the surrogate component selection criteria.
- Then, the formulation of this initial surrogate was adjusted in an iterative process to match target properties of the real fuel, including the hydrogen/carbon ratio, density, viscosity, vapor pressure, derived cetane number, and smoke point. When targeting the fuel composition (hydrocarbon class distribution and carbon number distribution within each class) and the hydrogen/carbon ratio, combustion properties such as the lower heating value and the heat capacity are easily matched as well, as results in the following section will show. However, other properties such as the vapor pressure, the smoke point, and the derived cetane number are more challenging to match and require further composition adjustments, so they have also been included as target in the surrogate formulation process. Aside from lower heating value, other fuel properties that have been evaluated but not used as targets in the surrogate formulation process include heat of vaporization, flash point, freezing point, heat capacity, surface tension, and distillation curve. However, these untargeted properties can be reproduced with reasonably good agreement when targeting the hydrocarbon class distribution and carbon number distribution within each hydrocarbon class, as will be demonstrated later.

For every surrogate candidate, properties were estimated with mixing rules based on the surrogate composition and characteristics of each individual species. Blending rules for ideal liquid mixtures were used for the carbon and hydrogen content, density, lower heating value, heat of vaporization, and heat capacity. The Lee–Keser equation^55^ was used to estimate the vapor pressure of the individual surrogate components, whereas Raoult’s law was used to calculate the vapor pressure of the surrogate (note that Raoult’s law typically does not work well with polar species such as ethanol, but all fuels tested in this paper have low polarity). Kendall–Monroe equation^56^ was used to estimate the kinematic viscosity, Thiele formula^57^ was used to obtain the flash point, freezing point was calculated using the freezing point blending index approach proposed in ref 58, Dalton mass-average equation^59^ was used for surface tension, and REFPROP^60^ was used to predict the distillation curve.

The smoke point of the surrogate candidates was calculated by volumetrically averaging the reciprocals of the smoke points of their individual components.^61^ The derived cetane number of the surrogate candidate was calculated following the method proposed in ref 62. The ignition delay of the most reactive mixture fraction of a spray obtained from a 1D spray model^63^ at conditions of the derived cetane number test in an ignition quality tester is correlated with the cetane number of the fuel. The correlation was constructed based on simulations of multiple fuels and fuel blends with known cetane numbers and showed a root-mean-square error of 3 cetane units. The same detailed chemical kinetic mechanism validated in the previous subsection was used for the prediction of the derived cetane number.

## 3 Results and Discussion

This section includes the presentation and analysis of the results derived from the study with the CRN. First, the resulting formulation from the modeling methodology presented in previous sections is shown and briefly discussed. After that, the validation of the CRN modeling workflow for an aeroengine with Jet-A is presented in Section 3.2. This study makes up a sort of baseline case, from which fuel effects are investigated: in Section 3.3—a variation of fuel from Jet-A to three different SAFs and in Section 3.4—the substitution of aromatics with cycloalkanes.

### 3.1 Fuel Surrogate Results

Surrogate fuels for Jet-A and for three fuels defined within the NJFCP were developed, namely:

- A2 (Jet-A with POSF#10352).
- C1 (alcohol-to-jet fuel with low cetane number from Gevo with POSF#11498).
- IPK (Fischer–Tropsch iso-paraffinic kerosene from Sasol with POSF#5642).
- C4 (a blend of 40%_vol_ C1 and 60%_vol_ IPK with POSF#12344).

Table 3 shows the derived composition of the surrogate fuels following the methodology in Section 2.3.

**Table 3:** Composition of the Surrogate Fuels for Jet-A, C1, IPK, and C4 (Liquid Volume Fractions)

parameter	A2–Jet-A	C1–ATJ	IPK	C4
*N*-tridecane	26.71%			
2,2,5-trimethylhexane			8.29%	5.86%
2-methylnonane			38.16%	25.80%
2,2,4,6,6-pentamethyl heptane	31.97%	83.58%	53.17%	63.02%
2,2,4,4,6,8,8-heptamethyl nonane		16.42%		5.32%
butylcyclohexane	28.22%			
1,2,4-trimethylbenzene	9.08%			
α-methylnaphtalene	4.02%			

To assess the representativity of the derived surrogates, Table 4 compares their main properties (predicted according to the previously defined methodology) to those of the real fuels (measured values from ref 64 following ASTM standards).[Fn fn1] In general, the surrogates reproduce not only the target properties of the real fuels (hydrogen/carbon ratio, density, viscosity, vapor pressure, derived cetane number, and smoke point) but also other properties that may be important for jet fuel (lower heating value, heat of vaporization, flash point, freezing point, heat capacity, surface tension, and distillation curve). Figure 6 shows the variation against temperature of density, kinematic viscosity, and vapor pressure for both real fuels and their surrogates.

**Table 4:** Main Properties of the Surrogate Fuels Compared to Those of the Real Fuels

	A2–Jet-A			C1–ATJ			IPK			C4		
parameter	fuel	surrog.	(error %)	fuel	surrog.	(error %)	fuel	surrog.	(error %)	fuel	surrog.	(error %)
C	11.4	11.13	2.4	12.5	12.54	–0.3	10.8	10.88	–0.7	11.4	11.38	0.2
H	21.7	21.79	–0.4	27.1	27.08	0.1	23.6	23.77	–0.7	24.7	24.76	–0.2
C/H ratio [-]	1.90	1.96	–3.2	2.17	2.16	0.5	2.19	2.18	0.5	2.17	2.17	0.0
*A*/*F* stoichiometric ratio [-]	14.6	14.7	–0.7	14.9	15.0	–0.7	15.0	15.0	0.0	15.0	15.0	0.0
molecular weight [g/mol]	158.6	155.4	2.0	178.0	177.6	0.2	152.9	154.3	–0.9	162.2	161.3	0.6
lower heating value [MJ/kg]	43.06	43.32	–0.6	43.88	44.09	–0.5	41.57	44.12	–6.1	43.79	44.11	–0.7
heat of vaporization [MJ/kg]	0.36	0.342	5.0	0.35	0.278	20.6	0.277	0.307	–10.8	0.35	0.298	14.9
flash point [°C]	48	51.1	–6.5	49.5	62.7	–26.7	53	47.5	10.4	44.5	51.8	–16.4
freezing point [°C]	–51	–50.2	1.6	–61	–60.3	1.1	<−65	–73.6	–13.2	–61	–69.7	–14.3
surface tension at 40 °C [dyn/cm]	23.6	24.1	–2.1	21.0	20.9	0.5	20.3	20.4	–0.5	21.1	20.6	2.4
heat capacity at 40 °C [kJ/kgK]	2.07	2.06	0.5	2.03	2.04	–0.5	2.16	2.19	–1.4	2.18	2.17	0.5
T10 [°C]	176.8	164.6	6.9	178.9	181.3	–1.3	181.5	159.1	12.3	169.4	164.6	2.8
T50 [°C]	205.4	179.4	12.7	183.3	181.4	1.0	188.6	187.0	0.8	179.7	179.3	0.2
T90 [°C]	244.6	209.0	14.6	224.4	220.9	1.6	209.8	194.5	7.3	206.5	209.0	–1.2
smoke point [mm]	22	23.9	–8.6	34.5	34.4	0.3	42	37.1	11.7	37.2	36.2	2.7
derived cetane number [-]	48.3	46.9	2.9	16.0	15.8	1.3	31.3	31.1	0.6	28	27.1	3.2

**Figure 6:**
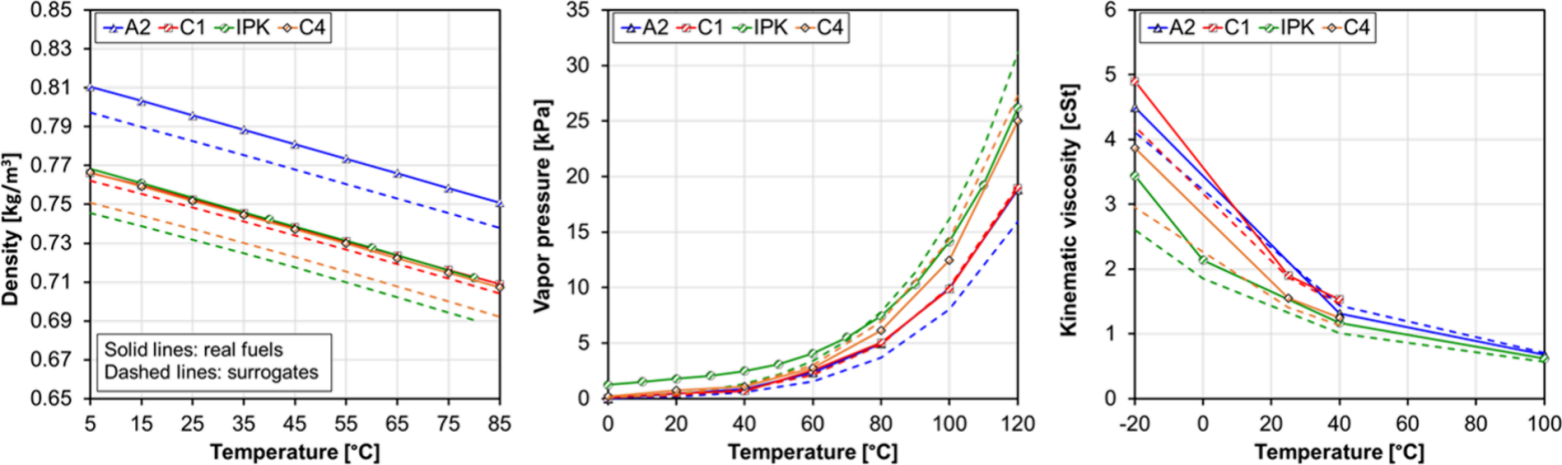
Density (left), kinematic viscosity (middle), and vapor pressure (right) against temperature for the real fuels (solid lines) and their surrogates (dashed lines). Symbols correspond to measured values for the real fuels.

While the previous comparison dealt mainly with fuel properties, additional validation of the surrogate fuels in more representative combustion situations has been performed by comparing ignition delay and laminar flame speed simulations. Figure 7 shows ignition delay data against temperature for Jet-A (top), C1 (middle), and IPK (bottom), where experiments with the real fuels are represented by symbols and simulations with the surrogate fuels are represented by lines. More specifically, shock tube ignition delay data from Wang et al.,^65^ Kim et al.,^66^ Min,^67^ Lee et al.,^68^ Zhu et al.,^69^ Richter et al.,^48^ Wang and Oehlschlaeger,^70^ and Lee^71^ were simulated. Figure 8 shows laminar flame speed measurements for Jet-A (top, experiments of Kun et al.^65^ and Rui et al.^72^), C1 (middle, experiments of Kun et al.^65^ and Richter et al.^48^) and IPK (bottom, experiments of Won et al.^73^) and the corresponding simulations with the surrogates described in Table 3. Equivalence ratio sweeps at different temperatures and pressure conditions were simulated, and the numerical results show good performance for all fuels.

**Figure 7:**
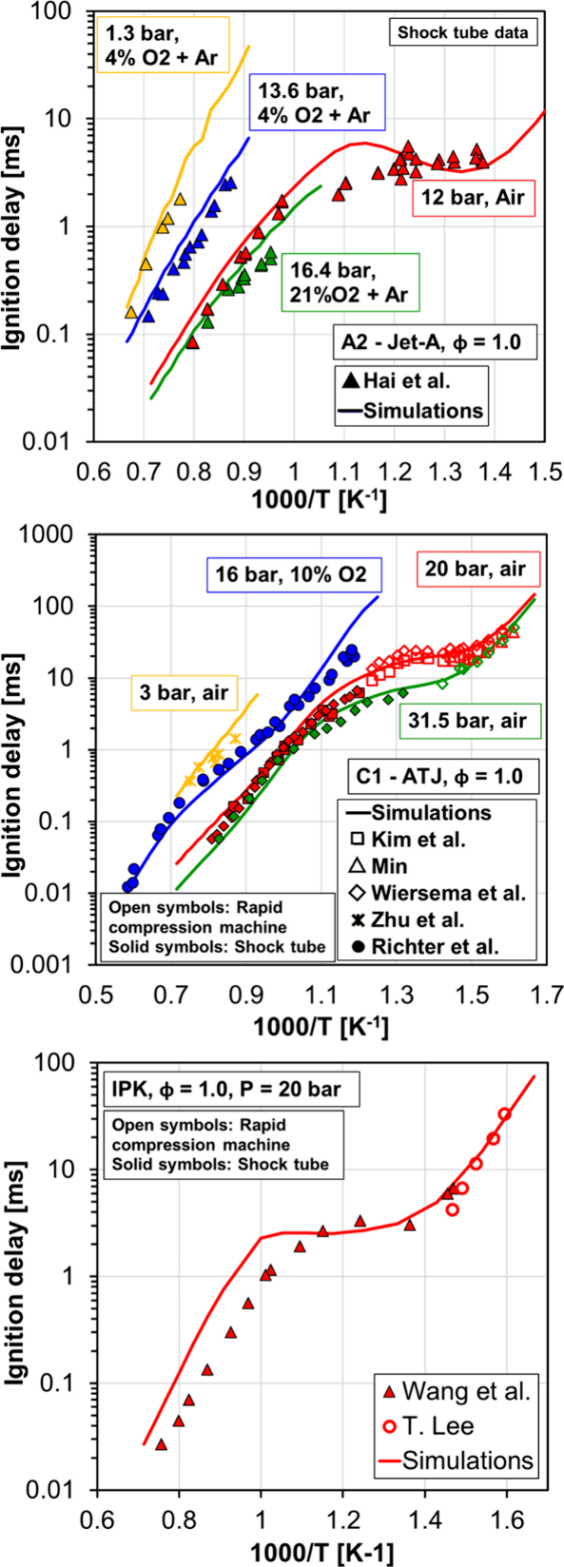
Ignition delay vs temperature for Jet-A (top), C1 (middle), and IPK (bottom). Experiments are represented by symbols, and simulations are represented by lines.

**Figure 8:**
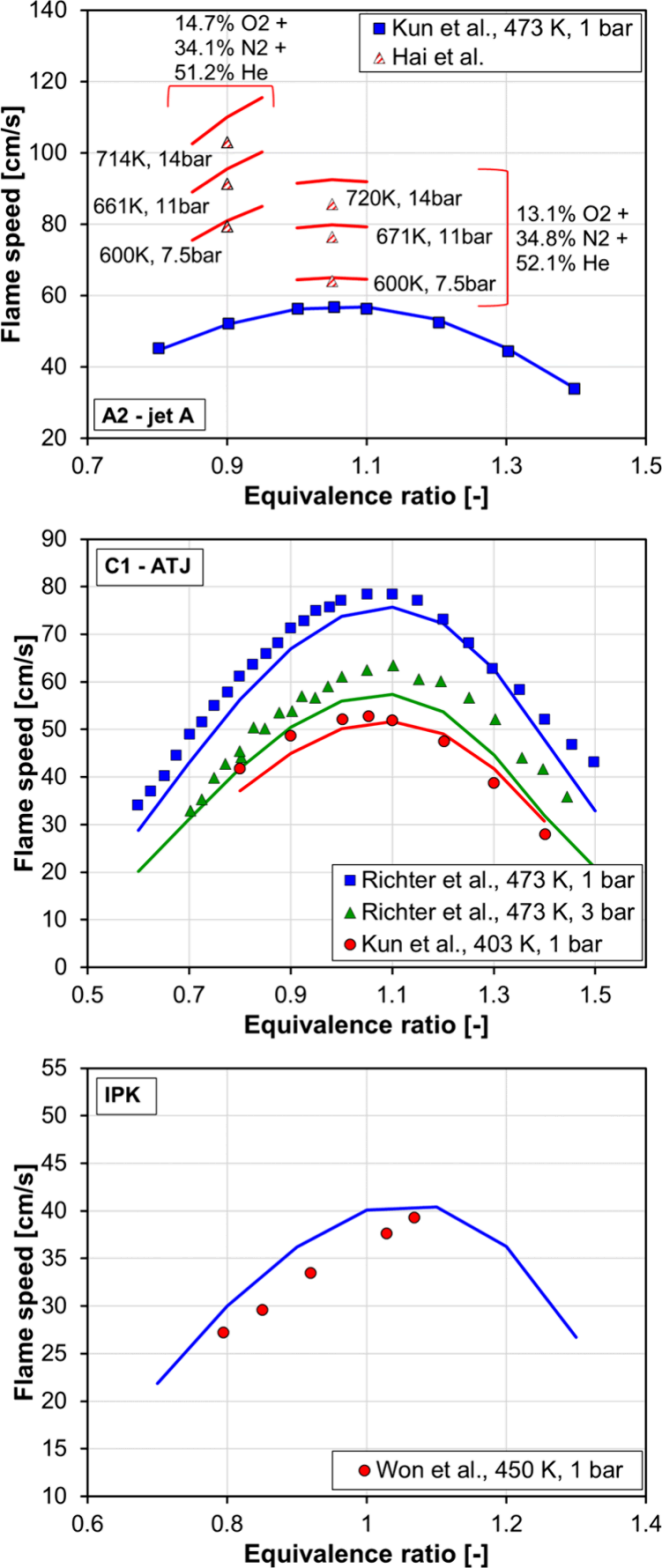
Laminar flame speed vs equivalence ratio for Jet-A (top), C1 (middle), and IPK (bottom). Experiments are represented by symbols, and simulations are represented by lines.

Results presented in Figures 7 and 8 demonstrate that the combination of the detailed chemical kinetic mechanism with the surrogate fuels proposed here can reproduce the combustion characteristics of Jet-A, C1, and IPK with good accuracy, providing a numerical tool for further analysis of these fuels. Unfortunately, the authors could not find experimental measurements of C4 in the literature. However, C4 is a blend of 40% C1 and 60% IPK and the chemical kinetic mechanism used here has been shown to work well for fuel blends, which gives confidence in the numerical tool. Nevertheless, additional ignition delay and laminar flame speed validations with C4 are recommended with data availability.

### 3.2 Validation of the Aeroengine CRN Modeling Workflow with Jet-A

The model validation includes the previously formulated CRN with the operating conditions for the CFM56 7B27/B1F engine fueled with Jet-A presented by Saboohi et al. in ref^41^ and compared with the emission indices from the Aircraft Engine Emissions Databank from the International Civil Aviation Organization (ICAO).^74^ These relate to the flight phases (takeoff, climb, approach, and idle at 100, 85, 30, and 7% available thrust, respectively) where engine emissions significantly impact the atmospheric environment and, specifically, the air quality around airports.

Figure 9 shows the validation results for the combustor exhaust temperature, the CO emission index (EICO), and the NO_*x*_ emission index (EINO_*x*_). The model accurately replicated the experimental exhaust temperature and the NO_*x*_ emissions. However, although the overall trend for CO emissions was accurate, results at approach were slightly overpredicted. CO is formed in the rich primary zone and partially oxidized in subsequent zones. At low loads, the combustor temperature is lower, leading to incomplete combustion, resulting in very low NO_*x*_ emissions but very high CO emissions. As the engine load increases, so do both the overall equivalence ratio and the temperature. Consequently, combustion becomes more complete and CO emissions decrease, while NO_*x*_ emissions increase.^36^ The overestimation of CO emissions in the model seems to be due to overly short residence times in the flame front region, suggesting that further model optimization could improve these results (e.g., by adjusting the flame front thickness).

**Figure 9:**
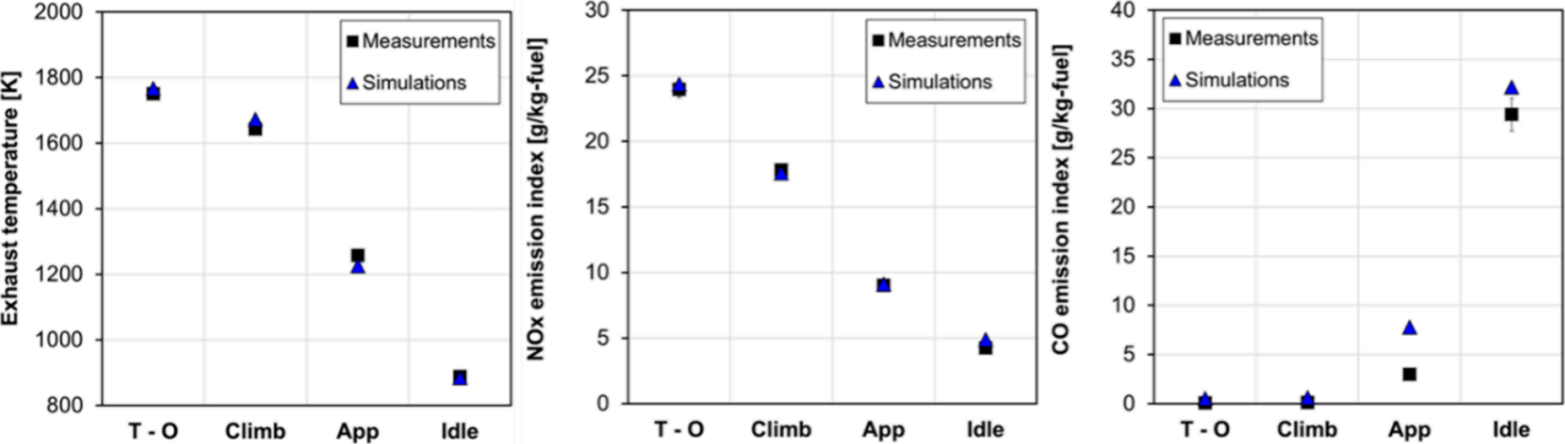
Comparison of measured (ICAO Databank) and simulated exhaust temperature (left), EINO_*x*_ (middle), and EICO (right) at various operating conditions; the fuel is Jet-A.

Figure 10 shows the concentration of the soot precursor cyclo-penta pyrene (A4R5), which could be considered some sort of surrogate for soot mass behavior. The current model includes only gas-phase chemistry and is not coupled with a soot formation model. However, the emission index of soot precursor A4R5 at the exhaust follows the same trend as the experimental PM emission index (EIPM), which gives confidence in the ability of the model to provide meaningful trends for the engine PM emissions.

**Figure 10:**
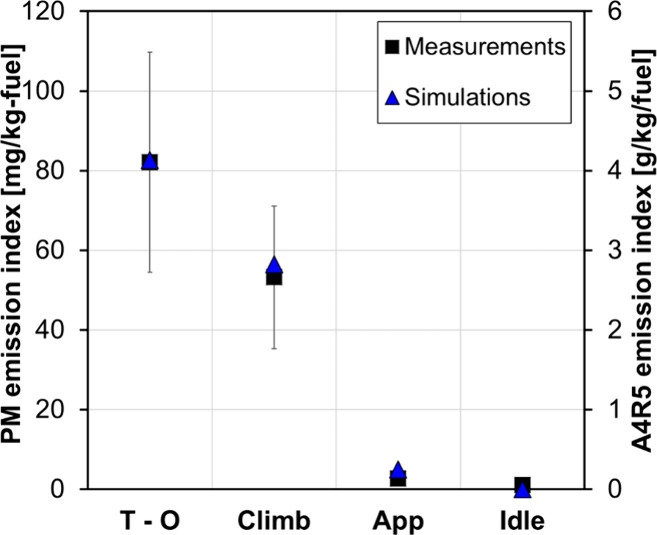
Comparison of soot measured (ICAO Databank) and simulated cyclo-penta pyrene at various operating conditions; the fuel is Jet-A.

### 3.3 Modeling Scenario 1: Combustion Performance of 100% SAF against Jet-A

In this section, Jet-A emissions are compared against those of the SAF surrogates described in Table 3. This situation mimics a theoretical scenario where 100% SAF is used, which is beyond current regulatory cap of SAF to 50%. The section will help quantitatively evaluate the departure of combustion behavior of SAF from that of Jet-A. The results for the combustor exhaust temperature, EINO_*x*_, and EICO are presented in Figure 11. Temperature and EINO_*x*_ exhibit similar values for all of the fuels utilized. This finding aligns with various experimental studies that have reported comparable NO_*x*_ emission behavior when using different types of SAFs such as HEFA and FT-SPK, compared to traditional Jet-A.^8,21,75^ These results reinforce the notion that NO_*x*_ emissions remain unaffected by the substitution of SAFs since the air/fuel stoichiometric ratio and the lower heating value, and hence the adiabatic flame temperature, do not change significantly among fuels. Regarding CO emissions, these results are very similar to those of Bulzan et al., who measured emissions at an APU using an FT fuel made from coal. In their study, the authors demonstrated that the use of pure FT fuel slightly decreased CO emissions at lower exhaust temperatures (idle), but the emissions were essentially identical at higher temperatures (takeoff).^21^ Jet-A is more susceptible to producing CO compared to C1, IPK, and C4 due to its chemical composition, which contains 13.06% 1,2,4-trimethylbenzene and 5.68% α-methylnaphthalene. Both aromatic compounds with stable structures have an inhibiting effect on the chemistry of alkanes, which reduces the reactivity, leading to higher CO production. In contrast, C1, IPK, and C4 lack aromatic compounds, which facilitate more complete combustion and lower CO emissions.^76,77^

**Figure 11:**
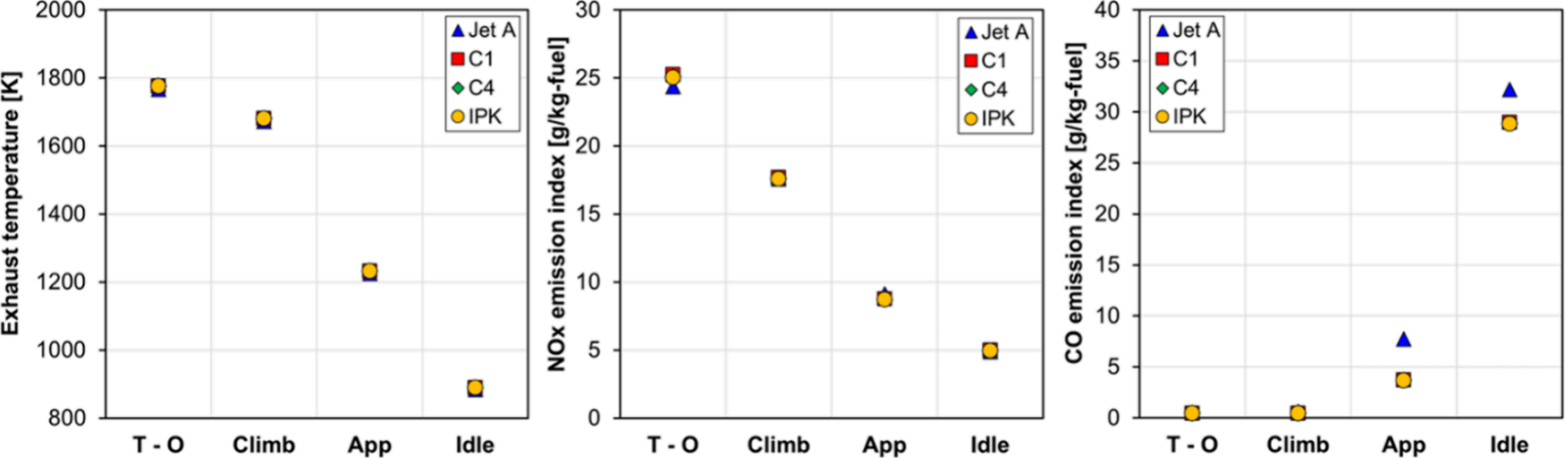
Simulated exhaust temperature (left), EINO_*x*_ (middle), and EICO (right) for all fuels under various operating conditions.

The differences in CO emissions between fuels can be further analyzed in the CRN. CO is primarily produced in the primary zone due to the rich global equivalence ratio and subsequently converted into CO_2_ as it reacts with available OH radicals mainly in the flame front and later in the downstream zones of the combustor. Interestingly, the amount of CO leaving the flame front and the intermediate zone is similar for all fuels, and the differences in the amounts of CO among fuels are controlled by the CO-to-CO_2_ oxidation reactions that occur in the dilution zone. Figure 12 illustrates the mole fractions of CO and OH entering the dilution zone (left) and the CO-to-CO_2_ oxidation rate within the dilution zone (right) for Jet-A and C1. As shown in the figure, the amount of CO that enters the dilution zone is virtually the same for both fuels. However, the concentration of OH radicals available to react with CO is 7.4% higher for C1, as this fuel is composed of iso-alkanes that promote the production of active radicals such as OH, helping achieve a more complete combustion compared to aromatic compounds.^78^ The resulting CO oxidation rate within the dilution zone is two times higher for C1 fuel, which results in lower CO emissions.

**Figure 12:**
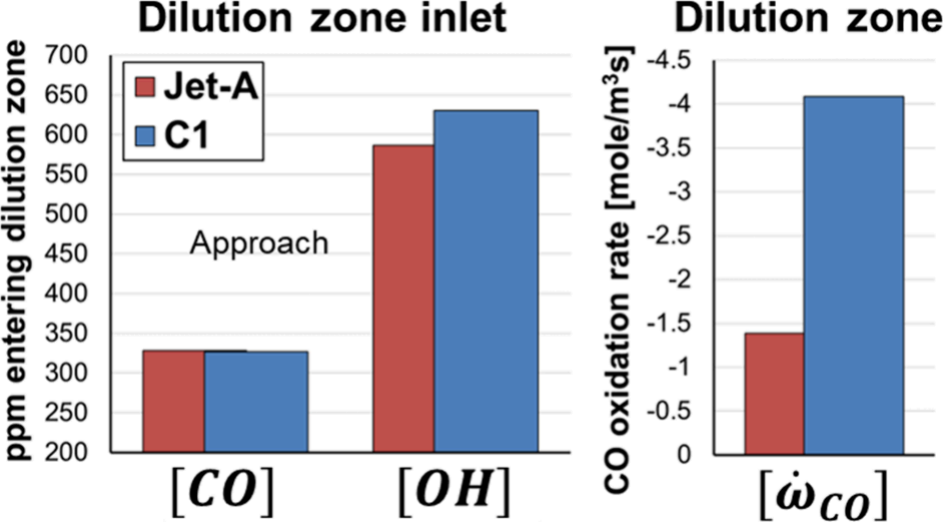
(Left) OH and CO available at the inlet of the dilution zone stemming from the intermediate zone. (Right) Oxidation rates for CO. Approach condition.

Cyclo-penta pyrene (A4R5), the largest PAH available in the chemical kinetic mechanism, was chosen as the reference species to analyze the behavior of PAHs in the model. Note that pyrenes are typically considered to be the soot precursors in many soot models. Figure 13 shows the A4R5 emission index for Jet-A and for the SAF surrogates tested in this study under the different operating conditions. A4R5 emissions decrease by 93% during takeoff when using 100% SAF compared to Jet-A, and similar reductions were observed across all conditions. Figure 14 shows the evolution of the mole fraction of A4R5 along the different zones within the combustor for Jet-A and C1 at the climb condition. In both cases, A4R5 forms in the rich primary zone and the mole fraction of A4R5 decreases in the subsequent zones due to air entrainment from the wall holes of the combustor. Thus, model predictions indicate that PAH emissions are almost exclusively controlled by the formation rate of soot precursors in the primary zone, suggesting that reduced residence times and high local equivalence ratios in the primary zone are key to reduce soot emissions. These results are in good agreement with those of Cain et al., who show that a low aromatic content in the fuel decreases benzene and toluene levels in the reaction zone and, therefore, the formation of large PAHs and, consequently, soot.^22^

**Figure 13:**
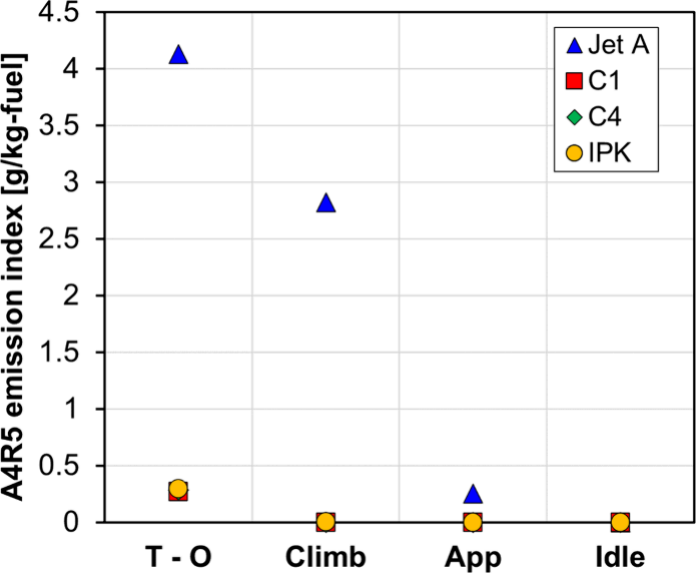
EIA4R5 for all fuels and operating conditions.

**Figure 14:**
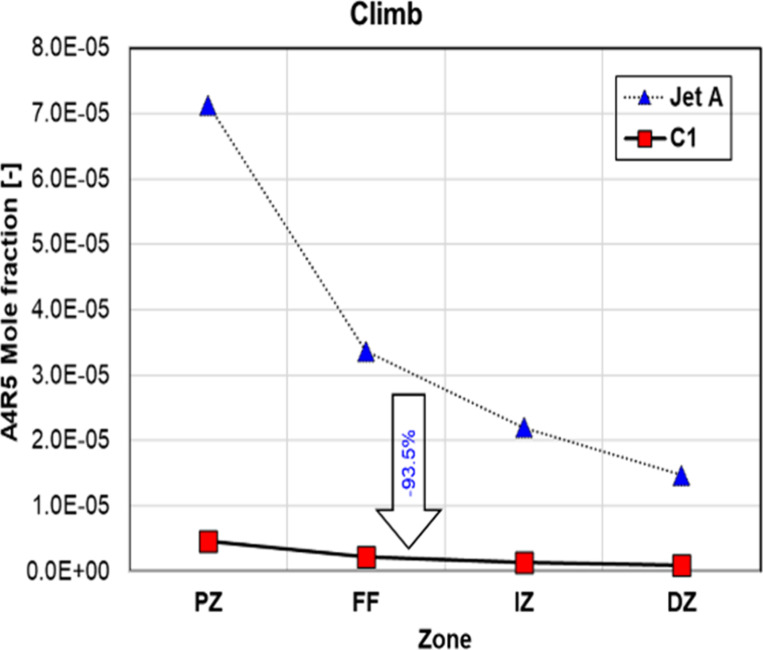
Evolution of A4R5 within the combustor for Jet-A and C1 at climb.

The previous results have been obtained for a scenario of neat SAF use in an aeroengine, which will probably be a future situation. From a current perspective, it is also interesting to gain further insight into the effect of an increasing proportion of SAF in Jet-A. For that purpose, blends of Jet-A and C1 have been simulated in the CRN, and the corresponding CO emission index and cyclo-penta pyrene mole fraction at combustor outlet are shown in Figure 15. Cyclo-penta pyrene emissions are shown at takeoff conditions because this represents a worst-case scenario for soot emissions, whereas CO emissions are shown at approach conditions because EICO is virtually zero at takeoff. To get a reference for comparison, a linear blending behavior is represented by the gray dashed lines. Results show a nonlinear blending effect on CO and A4R5 emissions, with a stronger-than-linear effect of C1 on decreasing CO and a weaker-than-linear effect on decreasing PAH emissions. The latter effect can be explained due to the high sensitivity of PAH formation to the presence of diaromatics in the fuel.

**Figure 15:**
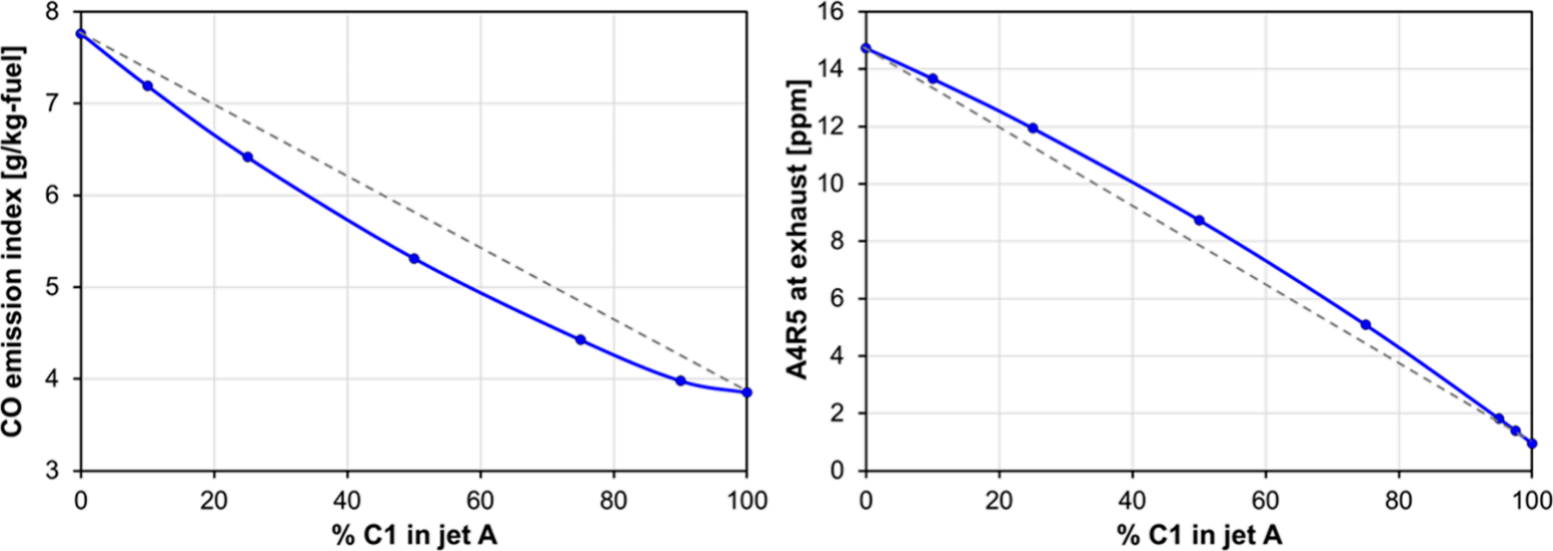
Evolution of combustor-outlet CO at approach and A4R5 at takeoff for binary blends of Jet-A and C1.

### 3.4 Modeling Scenario 2: Combustion Performance of Jet-A with Cycloalkane Substitution

Confronting the close link between aromatics and soot production, ASTM D7566 ^27^ jet fuel requests a minimum proportion of aromatic compounds to ensure sufficient seal swelling in aircraft and fuel circulation systems. In this direction, Kosir et al.^79^ proved the potential of cycloalkanes to provide the seal-swelling characteristics required for aviation fuel. However, Wu et al. demonstrated that replacing the aromatic content with cycloalkanes can also significantly influence combustion behavior, which is relevant when assessing high-ratio SAF substitution strategies.^29^ Such results have motivated a second scenario to explore the effect of replacing aromatic compounds within Jet-A with cycloalkanes, which aims to reduce PAH production. Figure 16 summarizes the six different substitution cases that have been investigated based on two directions:

- Three cycloalkane species are considered: *n*-butyl cyclohexane (NBCH), which is already a major component of the Jet-A surrogate, decahydronaphthalene (Decalin), and cyclohexane (CHX). These cycloalkanes have been selected based on availability in the chemical kinetic mechanism and because they represent three different molecular structures within the cycloalkane class: a bicyclic molecule, a branched molecule, and a nonbranched ring.
- For situations within case A, only the surrogate fraction corresponding to diaromatics (α-methylnaphthalene) is replaced by a corresponding single cycloalkane, while in case B, all aromatic components (α-methylnaphthalene and 1,2,4-trimethylbenzene) are replaced by the corresponding cycloalkane.

**Figure 16:**
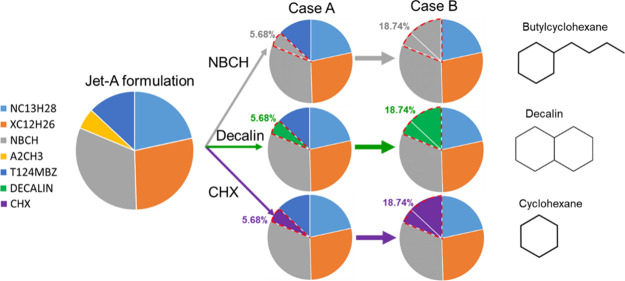
Schematic of cases dealing with the replacement of diaromatics and alkylbenzenes of Jet-A by cycloalkanes. Pie charts represent mole fraction.

Finally, it is important to consider the impact of replacing aromatics with cycloalkanes on the properties of the fuel, especially on those regulated in the ASTM D1655 standard, which, to some extent, will confirm if the corresponding fuel would be certified. This issue has been evaluated in Table 5 using the same blending rules as for the formulation of surrogate fuels (Section 2.3). Overall, fuel properties do not change significantly when aromatics are replaced with NBCH or Decalin. Only when CHX is used are significant differences observed, namely, both vapor pressure and viscosity increase and flash point decreases, so the selected fuels would be mostly within the specifications of typical kerosene.

**Table 5:** Key ASTM D1655 Properties of Jet-A with Cycloalkane Substitution

	Jet-A	NBCH		Decalin		CHX	
parameter	baseline	case A	case B	case A	case B	case A	case B
lower heating value [MJ/kg]	43.32	43.55	43.79	43.51	43.65	43.56	43.83
flash point [°C]	51.1	50.8	50.2	50.7	50.5	45.1	35.3
freezing point [°C]	–50.2	–52.3	–55.4	–50.2	–48.6	–49.3	–45.5
vapor pressure at 40 °C [kPa]	0.565	0.568	0.569	0.558	0.544	1.939	4.996
viscosity at –20 °C [cSt]	4.118	3.844	3.541	4.013	4.008	4.804	7.381

Figure 17 shows values of the A4R5 mole fraction at the combustor outlet (left), CO emissions index (middle), and NO_*x*_ emissions index (right) for Jet-A and for all six cases. Following a similar approach as in the previous section, the takeoff condition was selected for the A4R5 comparison, and the approach condition was selected for the CO comparison. NO_*x*_ results are shown at both takeoff and approach for completeness. Case A shows a 92% reduction in A4R5 emissions compared to Jet-A, and further replacement of all aromatics (case B) leads to an even greater reduction (95%). Similar results are obtained independently of the cycloalkane species. These results confirm that the production of soot precursors may be dramatically reduced by replacing only the diaromatics of the fuel while keeping the alkyl benzenes for seal swelling.^28^

**Figure 17:**
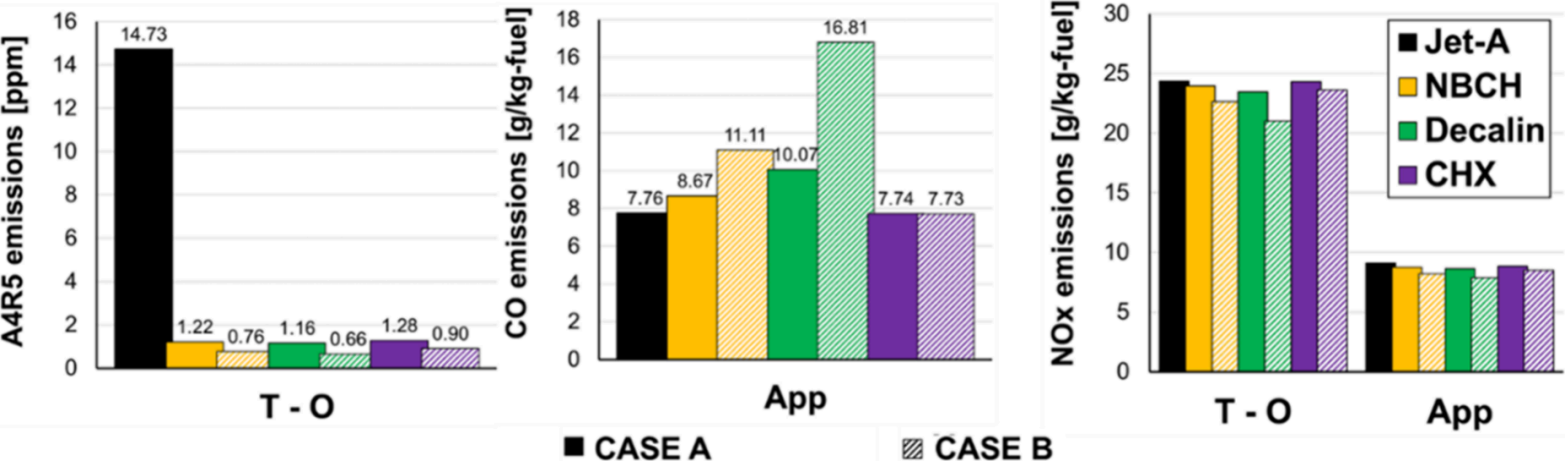
Simulated PAH (left), CO (middle), and NO_*x*_ (right) emissions with cycloalkanes replacing aromatics in Jet-A. Case A (solid bars) corresponds to replacement only of diaromatics. Case B (dashed bars) corresponds to full replacement of all of the aromatics.

CO emission shows that cycloalkane substitution with *n*-butyl cyclohexane and Decalin leads to an increase in the CO emission index, with the effect being worse for Decalin, whereas CO emissions do not change when aromatics are replaced with cyclohexane. Chemical kinetic analyses of CO formation indicate that this is caused by a slower burning rate in the primary zone with *n*-butyl cyclohexane and Decalin, which results in lower combustion temperature and slower oxidation of CO to CO_2_. To confirm this argument, Figure 18 shows the evolution of the temperature for the different zones within the combustor for case B with Decalin. The temperature in the primary zone is more than 100 K lower for Decalin, leading to higher CO formation. This inversely impacts NO_*x*_ formation, with cases having higher CO emissions showing lower NO_*x*_ emissions (Figure 17, right) indicating a strong trade-off between CO and NO_*x*_.

**Figure 18:**
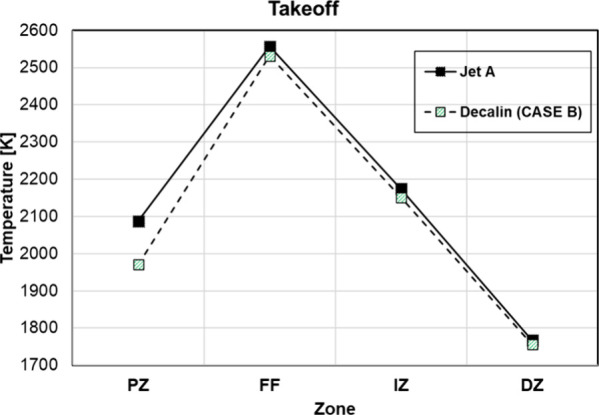
Evolution of temperature within the combustor for Decalin replacing all aromatics in Jet-A.

In summary, despite the strong potential of cycloalkanes to reduce soot emissions of jet fuel, combustion performance and resulting fuel properties according to standards show different sensitivities depending on the particular species. Therefore, careful selection of the aromatic replacement species has to be carried out, bringing in also the capability of achieving a comparable level of seal swelling to that of the original jet fuel.^80^

## 4 Conclusions

A chemical kinetic mechanism and SAF surrogate were presented and integrated into a CRN that simulates the combustor of a CFM56 engine; the resulting model was validated against experimental data using surrogate fuels for Jet-A. The validated model was then used to investigate the effects of varying fuel composition on the exhaust emissions, both in terms of a transition from conventional fuel to SAF, as well as in terms of the fuel aromatic content.

The chemical kinetic mechanism demonstrated high accuracy in predicting the combustion characteristics of iso-cetane and iso-dodecane, which are key compounds in sustainable fuels and advanced biofuels, with validation performed against ignition delay, laminar flame speed, and extinction strain rate data from the literature. Surrogate fuels for Jet-A and three alternative SAF-like fuels from the NJFCP (C1, IPK and C4) were formulated to match key properties of their real counterparts, including the hydrogen/carbon ratio, density, viscosity, vapor pressure, derived cetane number, and smoke point. The surrogate fuels were then validated against ignition delay and laminar flame speed data from the literature using the detailed mechanism mentioned above.

The mechanism and surrogates were integrated into a CRN model composed of six PSR that simulates different zones of the combustor of the CFM56 engine. The size of each reactor was defined based on geometric data of the combustor, and the flow distribution in the different reactors was adjusted to closely match fuel and temperature distributions for each zone from CFD results from the literature. Then, the CRN modeling tool was validated against experimental data with Jet-A from the ICAO Databank at takeoff, climb, approach, and idle conditions. The model showed very good agreement with the experiments for combustor-out temperature and CO and NO_*x*_ emissions indices, and the emissions of cyclo-penta pyrene, the largest PAH in the mechanism, closely follow experimental soot emissions trends.

Simulations with Jet-A, C1, IPK, and C4 demonstrated that SAF significantly reduces PAH emissions compared to Jet-A, with a 93% decrease in cyclo-penta pyrene emissions during takeoff. This aligns with studies showing that reducing aromatic compounds in fuel lowers emissions of soot precursors. Moreover, SAF shows lower CO emissions than Jet-A with no penalty for NO_*x*_ emissions. This is because of a higher CO-to-CO_2_ oxidation rate in the dilution zone with SAF thanks to a higher formation of OH radicals during the combustion process.

Replacing aromatic compounds in Jet-A with cycloalkanes led to a substantial reduction in PAH emissions (up to 95% in some cases). However, this substitution may also result in increased CO emissions due to a slower burning rate and lower flame temperature of some cycloalkane species compared to aromatics, and a clear trade-off between CO and NO_*x*_ emissions has been identified from the simulations. Finally, the cycloalkane species considered as a substitution for aromatics should be carefully selected to ensure the fuel meets property standards.

## Supplementary Material



## Footnotes

† Measurement methods for the properties reported in Table 3 include: GCxGC for molecular formula and derived properties, D4052 for density, D445 for viscosity, D4809 for lower heating value, D93 for flash point, D5972 for freeze point, D1331A for surface tension, E1269 for heat capacity, D86 for distillation, D1322 for smoke point, and D6890 for derived cetane number.

## Notes

Air-flow distribution and interzone flow-splitting parameters used for calibration of the chemical reactor network model of the CFM56 7B27/B1F engine, along with the geometric characteristics of each reactor zone for all LTO operating conditions (PDF)

## Abbreviations

A4R5: cyclopentapyrene (soot precursor)
ASTM: American Society for Testing and Materials
CFM56: common turbofan engine model used in aviation
CO: carbon monoxide
CRN: chemical reactor network
DTBP: di-*tert*-butyl peroxide
EICO: emission index of carbon monoxide
EINO_*x*_: emission index of nitrogen oxides
EIA4R5: emission index of cyclopentapyrene (mg/kg fuel)
FT-SPK: Fischer–Tropsch synthetic paraffinic kerosene
GCxGC: comprehensive two-dimensional gas chromatography
HEFA: hydroprocessed esters and fatty acids
ICAO: International Civil Aviation Organization
LHV: lower heating value
NJFCP: National Jet Fuels Combustion Program
NO_*x*_: nitrogen oxides
PAH: polycyclic aromatic hydrocarbons
PSR: perfectly stirred reactor
SAF: sustainable aviation fuel
T10 T50 T90: distillation temperature at 10, 50, and 90% evaporation

## Nomenclatures and Units

ρ: density (kg/m^3^)
φ: equivalence ratio (dimensionless)
σ: surface tension (dyn/cm)
μ: viscosity (cSt)
*A*/*F*: air–fuel ratio (kg air/kg fuel)
CN: cetane number (dimensionless)
*L*: length (m)
*P*: pressure (Pa)
*T*: temperature (K)
*U*: flow velocity (m/s)

## References

[1] Federal Aviation Administration (FAA), “Aviation Climate Action Plan,” Nov. 2021.

[2] World Economic Forum, “Pathways to net-zero emissions from aviation.” Accessed: Feb. 12, 2025. [Online]. Available: https://www.weforum.org/agenda/2022/12/aviation-net-zero-emissions/.

[3] International Air Transport Association (IATA), “IATA Press Release.” Accessed: Feb. 12, 2025. [Online]. Available: https://www.iata.org/en/pressroom/2022-releases/2022-10-07-01/.

[4] ITF (2023), “Sustainable Aviation Fuels Policy Status Report Case-Specific Policy Analysis.” Accessed: Jan. 21, 2026. [Online]. Available: https://www.itf-oecd.org/sites/default/files/docs/sustainable-aviation-fuels-policy-status-report.pdf.

[5] Koscakova, M. ; Korba, P. ; Sekelova, I. ; Koscak, P. ; Pastir, D. , “Analysis of Sustainable Aviation Fuels Market,” in 2022 New Trends in Aviation Development (NTAD); IEEE, Nov. 2022; pp 123–127. 10.1109/NTAD57912.2022.10013491.

[6] Andrejczyk, R. , “Implications of Sustainable Aviation Fuel for the Rotorcraft Industry,” in Proceedings of the Vertical Flight Society 79th Annual Forum; The Vertical Flight Society, May 2023; pp 1–10. 10.4050/F-0079-2023-18146.

[7] Jiang C., Yang H. (2021). Carbon tax or sustainable aviation
fuel quota. Energy Econ..

[8] Schripp T. (2022). Aircraft engine particulate
matter emissions from sustainable aviation
fuels: Results from ground-based measurements during the NASA/DLR
campaign ECLIF2/ND-MAX. Fuel.

[9] Teoh R. (2022). Targeted Use of Sustainable
Aviation Fuel to Maximize Climate Benefits. Environ. Sci. Technol..

[10] NASA, “NASA, Boeing Gather Data to Aid Sustainable Aviation Fuel Adoption.” Accessed: Feb. 12, 2025. [Online]. Available: https://www.nasa.gov/centers-and-facilities/langley/nasa-boeing-gather-data-to-aid-sustainable-aviation-fuel-adoption-2/.

[11] Sustainable Aviation Buyers Alliance, “Sustainable Aviation Buyers Alliance Announces Aviation Decarbonization First, with Collective Purchase of Sustainable Aviation Fuel Certificates.” Accessed: Jan. 21, 2026. [Online]. Available: https://newsroom.bankofamerica.com/content/newsroom/press-releases/2023/04/sustainable-aviation-buyers-alliance-announces-aviation-decarbon.html.

[12] U.S. Department of Energy, “Sustainable Aviation Fuels.” Accessed: Feb. 12, 2025. [Online]. Available: https://www.energy.gov/eere/bioenergy/sustainable-aviation-fuels.

[13] Gettelman A., Chen C.-C., Jacobson M. Z., Cameron M. A., Wuebbles D. J., Khodayari A. (2017). Coupled Chemistry-Climate
Effects from 2050 Projected
Aviation Emissions. Atmos. Chem. Phys. Disc..

[14] Colket M. (2017). Overview of the National Jet Fuels Combustion Program. AIAA J..

[15] Heyne, J. S. Year 3 of the National Jet Fuels Combustion Program: Practical and Scientific Impacts of Alternative Jet Fuel Research,” in 2018 AIAA Aerospace Sciences Meeting; American Institute of Aeronautics and Astronautics: Reston, VA, Jan. 2018. 10.2514/6.2018-1667.

[16] Christie S., Lobo P., Lee D., Raper D. (2017). Gas Turbine
Engine
Nonvolatile Particulate Matter Mass Emissions: Correlation with Smoke
Number for Conventional and Alternative Fuel Blends. Environ. Sci. Technol..

[17] Corporan, E. ; Monroig, O. ; Wagner, M. ; Dewitt, M. J. , “Influence of Fuel Chemical Composition on Particulate Matter Emissions of a Turbine Engine,” in Volume 1: Turbo Expo 2004; ASMEDC, Jan. 2004; pp 837–848. 10.1115/GT2004-54335.

[18] Xing, J. Y. ; Groth, C. P. , “Assessment of PAH-Based Precursor Models Using a Seven-Moment Quadrature-Based Closure for Soot Formation Prediction in Non-Premixed Laminar Flames,” in AIAA SCITECH 2022 Forum; American Institute of Aeronautics and Astronautics: Reston, VA, Jan. 2022. 10.2514/6.2022-2253.

[19] Richter S., Kathrotia T., Naumann C., Scheuermann S., Riedel U. (2021). Investigation of the sooting propensity of aviation
fuel mixtures. CEAS Aeronaut. J..

[20] Frenklach M. (2002). Reaction mechanism
of soot formation in flames. Phys. Chem. Chem.
Phys..

[21] Bulzan, D. “Gaseous and Particulate Emissions Results of the NASA Alternative Aviation Fuel Experiment (AAFEX),” in Volume 2: Combustion, Fuels and Emissions, Parts A and B; ASMEDC Oct. 2010; pp 1195–1207. 10.1115/GT2010-23524.

[22] Cain J. (2013). Characterization of Gaseous and Particulate Emissions
From a Turboshaft
Engine Burning Conventional, Alternative, and Surrogate Fuels. Energy Fuels.

[23] Schripp T. (2018). Impact of Alternative
Jet Fuels on Engine Exhaust Composition During
the 2015 ECLIF Ground-Based Measurements Campaign. Environ. Sci. Technol..

[24] Corporan E. (2011). Chemical, Thermal Stability,
Seal Swell, and Emissions Studies of
Alternative Jet Fuels. Energy Fuels.

[25] Durdina L., Brem B. T., Elser M., Schönenberger D., Siegerist F., Anet J. G. (2021). Reduction of Nonvolatile
Particulate
Matter Emissions of a Commercial Turbofan Engine at the Ground Level
from the Use of a Sustainable Aviation Fuel Blend. Environ. Sci. Technol..

[26] Moniruzzaman C. G., Yu F. (2012). A 0D aircraft engine
emission model with detailed chemistry and soot
microphysics. Combust. Flame.

[27] ASTM International, “Standard Specification for Aviation Turbine Fuel Containing Synthesized Hydrocarbons,” West Conshohocken, PA,19428–2959. United States, May 2024. Accessed: May 27, 2024. [Online]. Available: https://compass.astm.org/document/?contentCode=ASTM%7CD7566-15%7Cen-US&proxycl=https%3A%2F%2Fsecure.astm.org&fromLogin=true.

[28] Kosir S., Stachler R., Heyne J., Hauck F. (2020). High-performance jet
fuel optimization and uncertainty analysis. Fuel.

[29] Wu Y. (2026). Surrogate fuel formulation
and performance-driven fuel modulation
for sustainable aviation fuels: Strategies to match combustion characteristics
of conventional jet fuel. Combust. Flame.

[30] Villette S., Adam D., Alexiou A., Aretakis N., Mathioudakis K. (2024). A Simplified
Chemical Reactor Network Approach for Aeroengine Combustion Chamber
Modeling and Preliminary Design. Aerospace.

[31] Khodayari H., Ommi F., Saboohi Z. (2020). A review on
the applications of the
chemical reactor network approach on the prediction of pollutant emissions. Aircraft Engineering and Aerospace Technology.

[32] Yi, J. ; Manin, J. ; Wan, K. ; Lopez Pintor, D. ; Nguyen, T. ; Dempsey, A. , Effect of Fuel Chemical Structure on Soot Formation in Sustainable Aviation Fuels, Energy & Propulsion Conference & Exhibition; SAE Technical Paper Nov. 2024. 10.4271/2024-01-4310.

[33] Starik A. M., Lebedev A. B., Savel’ev A. M., Titova N. S., Leyland P. (2013). Impact of
Operating Regime on Aviation Engine Emissions: Modeling Study. J. Propuls. Power.

[34] Bisson J., Seers P., Huegel M., Garnier F. (2016). Numerical Prediction
of Gaseous Aerosol Precursors and Particles in an Aircraft Engine. J. Propuls. Power.

[35] Saboohi Z., Ommi F. (2017). Emission prediction
in conceptual design of the aircraft engines
using augmented CRN. Aeronautical Journal.

[36] Zhang Q., Hai H., Li C., Wang Y., Zhang P., Wang X. (2020). Predictions
of NOx and CO emissions from a low-emission concentric staged combustor
for civil aeroengines. Proc. Inst. Mech. Eng.
G J. Aerosp. Eng..

[37] Garcia-Oliver, J. M. ; García, J. G. ; Corzo, B. M. ; Laboulais, J. N. ; Leyland, P. , “Development of a Reactor Network Model to Predict Pollutant Emissions from Aviation Gas Turbines,” in Proceedings of the XV Ibero-American Congress of Mechanical Engineering; Springer International Publishing: Cham, 2023; pp 191–197. 10.1007/978-3-031-38563-6_28.

[38] Nguyen T. H. (2019). Improved
Chemical Reactor Network Application for Predicting the Emission of
Nitrogen Oxides in a Lean Premixed Gas Turbine Combustor. Combust. Explos. Shock Waves.

[39] Novosselov, I. V. ; Malte, P. C. ; Yuan, S. ; Srinivasan, R. ; Lee, J. C. Y. , “Chemical Reactor Network Application to Emissions Prediction for Industial DLE Gas Turbine,” in Vol. 1: Combustion and Fuels, Education; ASMEDC Jan. 2006; pp 221–235. 10.1115/GT2006-90282.

[40] Park J., Nguyen T. H., Joung D., Huh K. Y., Lee M. C. (2013). Prediction
of NO *x* and CO Emissions from an Industrial Lean-Premixed
Gas Turbine Combustor Using a Chemical Reactor Network Model. Energy Fuels.

[41] Saboohi Z., Ommi F., Fakhrtabatabaei A. (2016). Development
of an Augmented Conceptual
Design Tool for Aircraft Gas Turbine Combustors. Int. J. Multiphys..

[42] Wang M. (2020). Autoignition of CRC diesel surrogates at low temperature combustion
conditions: Rapid compression machine experiments and modeling. Combust. Flame.

[43] Sarathy S. M. (2011). Comprehensive chemical
kinetic modeling of the oxidation of 2-methylalkanes
from C7 to C20. Combust. Flame.

[44] Kukkadapu G., Wagnon S. W., Pitz W. J., Hansen N. (2021). Identification of the
molecular-weight growth reaction network in counterflow flames of
the C3H4 isomers allene and propyne. Proceedings
of the Combustion Institute.

[45] Glarborg P., Miller J. A., Ruscic B., Klippenstein S. J. (2018). Modeling
nitrogen chemistry in combustion. Prog. Energy
Combust. Sci..

[46] Fang R. (2020). Fuel molecular structure effect on autoignition of highly branched
iso-alkanes at low-to-intermediate temperatures: Iso-octane versus
iso-dodecane. Combust. Flame.

[47] Guzman J., Kukkadapu G., Brezinsky K., Westbrook C. (2019). Experimental
and modeling study of the pyrolysis and oxidation of an iso-paraffinic
alcohol-to-jet fuel. Combust. Flame.

[48] Richter S. (2022). A combined experimental
and modeling study of combustion properties
of an isoparaffinic alcohol-to-jet fuel. Combust.
Flame.

[49] Won S. H. (2016). Combustion characteristics
of C4 iso-alkane oligomers: Experimental
characterization of iso-dodecane as a jet fuel surrogate component. Combust. Flame.

[50] Oehlschlaeger M. A., Steinberg J., Westbrook C. K., Pitz W. J. (2009). The autoignition
of iso-cetane at high to moderate temperatures and elevated pressures:
Shock tube experiments and kinetic modeling. Combust. Flame.

[51] Mao Y. (2019). The autoignition of
iso-dodecane in low to high temperature range:
An experimental and modeling study. Combust.
Flame.

[52] Li B., Zhang H., Egolfopoulos F. N. (2014). Laminar flame propagation of atmospheric
iso-cetane/air and decalin/air mixtures. Combust.
Flame.

[53] Cheng S. (2022). Replicating HCCI-like
autoignition behavior: What gasoline surrogate
fidelity is needed?. Applications in Energy
and Combustion Science.

[54] MacDonald, J. ; Lopez Pintor, D. ; Matsubara, N. ; Kitano, K. ; Yamada, R. , “Effects of Ethanol Blending on the Reactivity and Laminar Flame Speeds of Gasoline, Methanol-to-Gasoline, and Ethanol-to-Gasoline Surrogates,” WCX SAE World Congress Experience; SAE Technical Paper Apr. 2024. 10.4271/2024-01-2817.

[55] Lee B. I., Kesler M. G. (1975). A generalized thermodynamic correlation based on three-parameter
corresponding states. AIChE J..

[56] Kendall J., Monroe K. P. (1917). THE VISCOSITY OF
LIQUIDS. II. THE VISCOSITY-COMPOSITION
CURVE FOR IDEAL LIQUID MIXTURES. 1. J. Am. Chem.
Soc..

[57] Santos S. M., Nascimento D. C., Costa M. C., Neto A. M. B., Fregolente L. V. (2020). Flash point
prediction: Reviewing empirical models for hydrocarbons, petroleum
fraction, biodiesel, and blends. Fuel.

[58] Coburn, A. ; Yang, Z. ; Boehm, R. ; Heyne, J. S. , “Determination of a Freeze Point Blend Prediction Model for Jet Fuel Range Hydrocarbons,” in AIAA SCITECH 2022 Forum; American Institute of Aeronautics and Astronautics: Reston, VA, Jan. 2022. 10.2514/6.2022-2221.

[59] Mousavi N. S., Romero-Martínez A., Ramírez-Verduzco L. F. (2020). Predicting
the surface tension of mixtures of fatty acid ethyl esters and biodiesel
fuels using UNIFAC activity coefficients. Fluid
Phase Equilib..

[60] Lemmon, E. W. ; Bell, I. H. ; Huber, M. L. ; McLinden, M. O. , “NIST Standard Reference Database 23: Reference Fluid Thermodynamic and Transport Properties-REFPROP, Version 10.0, National Institute of Standards and Technology,” 2018, https://www.nist.gov/srd/refprop.

[61] Haas, F. M. ; Qin, A. ; Dryer, F. L. , “‘Virtual’ Smoke Point Determination of Alternative Aviation Kerosenes by Threshold Sooting Index TSI) Methods,” in 50th AIAA/ASME/SAE/ASEE Joint Propulsion Conference; American Institute of Aeronautics and Astronautics: Reston, VA, Jul. 2014. 10.2514/6.2014-3468.

[62] Bin-Khalid U., Lopez-Pintor D., Micó C., Lee S. (2024). Potential of 2-ethylhexyl
nitrate (EHN) and di-tert-butyl peroxide (DTBP) to enhance the cetane
number of ethanol, a detailed chemical kinetic study. Fuel.

[63] Pastor J. V., Garcia-Oliver J. M., Pastor J. M., Vera-Tudela W. (2015). ONE-DIMENSIONAL
DIESEL SPRAY MODELING OF MULTICOMPONENT FUELS. Atomization and Sprays.

[64] Edwards, J. T. “Reference Jet Fuels for Combustion Testing,” in 55th AIAA Aerospace Sciences Meeting; American Institute of Aeronautics and Astronautics: Reston, VA, Jan. 2017. 10.2514/6.2017-0146.

[65] Wang H. (2018). A physics-based approach to modeling real-fuel combustion chemistry
- I. Evidence from experiments, and thermodynamic, chemical kinetic
and statistical considerations. Combust. Flame.

[66] Kim, K. Data-Driven Combustion Kinetic Modeling Concept of Alternative Alcohol-to-Jet (ATJ) Fuel,” in AIAA Scitech 2021 Forum; American Institute of Aeronautics and Astronautics: Reston, VA, Jan. 2021. 10.2514/6.2021-1245.

[67] Min, K. “Autoignition study of next generation aviation fuels and components,” PhD Thesis, University of Illinois at Urbana: Champaign, Illinois, 2020.

[68] Kim K. (2024). Experimental and data-driven
chemical kinetic modeling study of alcohol-to-jet
(ATJ) synthetic biofuel for sustainable aviation fuels. Fuel.

[69] Zhu Y., Li S., Davidson D. F., Hanson R. K. (2015). Ignition delay times of conventional
and alternative fuels behind reflected shock waves. Proceedings of the Combustion Institute.

[70] Wang H., Oehlschlaeger M. A. (2012). Autoignition
studies of conventional and Fischer–Tropsch
jet fuels. Fuel.

[71] Lee, T. , “Ignition and Oxidation of Bio-Derived Future Navy Fuels,” Arlington VA, USA, Aug. 2013.

[72] Xu R. (2018). A physics-based approach to modeling real-fuel combustion
chemistry
– II. Reaction kinetic models of jet and rocket fuels. Combust. Flame.

[73] Won S. H. (2016). Predicting the global
combustion behaviors of petroleum-derived and
alternative jet fuels by simple fuel property measurements. Fuel.

[74] E. European Union Aviation Safety Agency, “ICAO Aircraft Engine Emissions Databank.” Accessed: May 24, 2024. [Online]. Available: https://www.easa.europa.eu/en/domains/environment/icao-aircraft-engine-emissions-databank.

[75] Okai, K. ; Fujiwara, H. ; Mizuno, T. , “Evaluation of Emission during Simulated in-Flight Conditions Based on Experimental Data with an RQL Combustor Model,” in AIAA SCITECH 2023 Forum; American Institute of Aeronautics and Astronautics: Reston, VA, Jan. 2023. 10.2514/6.2023-1326.

[76] Agosta A., Cernansky N. P., Miller D. L., Faravelli T., Ranzi E. (2004). Reference components
of jet fuels: kinetic modeling and experimental
results. Exp. Therm. Fluid Sci..

[77] Wilk R. D., Koert D. N., Cernansky N. P. (1989). Low-temperature
carbon monoxide formation
as a means of assessing the autoignition tendency of hydrocarbons
and hydrocarbon blends. Energy Fuels.

[78] Rao S., Zheng Z., Yang C. (2023). Effect of Cyclohexane on the Combustion
Characteristics of Multi-Component Gasoline Surrogate Fuels. Molecules.

[79] Kosir S., Heyne J., Graham J. (2020). A machine learning
framework for
drop-in volume swell characteristics of sustainable aviation fuel. Fuel.

[80] Landera A. (2022). Building Structure-Property Relationships of Cycloalkanes in Support
of Their Use in Sustainable Aviation Fuels. Front. Energy Res..

