# Supplementary material for: Fuel Effects on Aviation Engine Emissions: A Chemical Reactor Network Modeling Study
Source: Energy Fuels. 2026 Feb 20;40(9):4777–92. doi: 10.1021/acs.energyfuels.5c05727 (PMC12969266; doi:10.1021/acs.energyfuels.5c05727)
Supplement: Supplementary file 1 [file ef5c05727_si_001.pdf]

Supporting Information for Publication

Fuel Effects on Aviation Engine Emissions: A Chemical Reactor Network Modeling Study

AUTHOR NAMES

Dario Lopez-Pintor[1], James MacDonald[1],

Elkin Ramirez-Correa[2], Jose Maria Garcia-Oliver[2], Raul Payri[2], Pedro Marti Gomez-Aldaravi[2]*

Pénélope Leyland[3]

AUTHOR ADDRESS

[1]Sandia National Laboratories, Livermore, California, 94550, USA

[2]CMT- Clean Mobility & Thermofluids, Valencia, Valencia, 46022, España

[3]Advanced Engineering Design Solutions (AEDS), 1921 Martigny-Croix Switzerland, and Tribology and Interfacial Chemistry Group, IMX, EPFL, 1015 Lausanne, Switzerland.

CRN calibration parameters

Table S1. Air flow distribution and flow separation factors, where F_i denotes the fraction of the total air mass flow rate $\dot{m}_{air}$ that is introduced into a given zone $_i$, and ωi represents the fraction of a flow at the outlet of one reactor that is directed to another one.

Parameter	Description	Take-off	Climb	Approach	Idle
F_{PZ}	Air flow into primary zone	0.1875	0.1792	0.130	0.095
F_{FF}	Air flow into flame front	0.232	0.209	0.114	0.049
F_{IZ}	Air flow into intermediate zone	0.23	0.23	0.23	0.23
F_{WZ_1}	Air flow into wall zone #1	0.01	0.01	0.01	0.01
F_{WZ2}	Air flow into wall zone #2	0.29	0.29	0.29	0.29
F_{DZ}	Air flow into dilution zone	0.051	0.082	0.225	0.326
$1-\omega_1$	$\dot{m}$ between PZ and FF	0.98	0.98	0.98	0.98
ω_1	$\dot{m}$ between PZ and WZ	0.02	0.02	0.02	0.02
ω_2	$\dot{m}$ between WZ and IZ	0.8	0.8	0.8	0.8
$1-\omega_2$	$\dot{m}$ between WZ and WZ2	0.2	0.2	0.2	0.2

Table S2. Geometry of CRN zones.

**Geometry of CFM56 7B27/B1F**			
Zone	$L\ [m]$	$A\ [m^2]$	$V\ [m^3]$
PZ	0.05137	0.1816	0.0093288
FF	0.00173	0.1816	0.0003142
IZ	0.05970	0.2160	0.0128952
WZ	0.05970	0.0580	0.0034626
WZ2	0.06480	0.0580	0.0037584
DZ	0.06480	0.2880	0.0186624
